# FTY720 Protects Cardiac Microvessels of Diabetes: A Critical Role of S1P1/3 in Diabetic Heart Disease

**DOI:** 10.1371/journal.pone.0042900

**Published:** 2012-08-14

**Authors:** Zhiyong Yin, Linni Fan, Liping Wei, Haokao Gao, Rongqing Zhang, Ling Tao, Feng Cao, Haichang Wang

**Affiliations:** 1 Department of Cardiology, Xijing Hospital, The Fourth Military Medical University, Xi’an City, Shaanxi Province, China; 2 Department of Pathology, Xijing Hospital, The Fourth Military Medical University, Xi’an City, Shaanxi Province, China; 3 Department of Cardiology, Tianjin Union Medicine Center, Tianjin, China; Research Inst. of Environmental Med., Nagoya Univ., Japan

## Abstract

***Background:*** Diabetes is associated with an increased risk of cardiac microvascular disease. The mechanisms by which this damage occurs are unknown. However, research suggests that signaling through the sphingosine-1-phosphates receptor 1 and 3 (S1P1/3) by FTY720, a sphiongolipid drug that is structually similar to SIP, may play a role in the treatment on cardiac microvascular dysfunction in diabetes. We hypothesized that FTY720 might exert the cardioprotective effects of S1P1 and S1P3 viaprotein kinase C-beta (PKCβ II) signaling pathway.

***Methodology/Principal Findings:*** Transthoracic echocardiography was performed to detect the change of cardiac function. Scanning and transmission electron microscope with lanthanum tracer were used to determine microvascular ultrastructure and permeability *in vivo*. Apoptosis was detected by TUNEL and CD31 dual labeling in paraffin-embedded sections. Laser capture miscrodissection was used to assess cardiac micovascular endothelial cells (CMECs) *in vivo*. RT-PCR and Western blot analysis were used to determine the mRNA levels and protein expression of S1P1, S1P3, and PKCβ II. In the diabetic rats vs. controls, cardiac capillaries showed significantly higher density; CD31 positive endothelial cells were significantly reduced; the apoptosis index of cardiac endothlial cells was significantly higher. And FTY720 could increase the expressional level of S1P1 and boost S1P3 trasnslocation from membrane to nuclear, then ameliorate cardiac microvascular barrier impairment and pathologic angiogenesis induced by diabetes. In addition, overexpression of PKCβ II significantly decreased the protective effect of FTY720.

***Conclusions:*** Our study represents that the deregulation of S1P1 and S1P3 is an important signalresponsible for cardiac microvascular dysfunction in diabetes. FTY720 might be competent to serve as a potential therapeutic approach for diabetic heart disease through ameliorating cardiac microvascular barrier impairment and pathologic angiogenesis, which might be partly dependent on PKCβII-mediated signaling pathway.

## Introduction

The global epidemic of diabetes has led to growing numbers of secondary cardiovascular complications, which in turn, affect the progression and prognosis of diabetic patients. [1]–[3]. A growing body of evidence indicates a negative impact of diabetes on vascular, and in particular, microvascular endothelial cells [4]–[9] such as abnormal neovascularization, which conversely aggravate the progression of diabetes [10]. Our prior studies have also demonstrated that cardiac microvessels contribute primarily to diabetic cardiopathy [11], [12], and further support the key role of cardiac microvascular dysfunction in diabetic pathogenesis. Likewise, it is reported that the initiation of pathology associated with angiogenesis involves changes in vascular permeability driven by various angiogenic factors, such as vascular endothelial growth factor (VEGF) [13], [14]. The above evidence provide new theoretical basis for the therapy targeting on cardiac microvessels. However, there still lack effective agents for such therapy.

Sphingosine-1-phosphate (S1P), a bioactive sphingolipic metabolite of platelets, is a potential proangiogenic molecule that acts by binding to five S1P receptors (S1P1–S1P5) [15]. Especially, the subtypes of S1P1 and S1P3 are expressed on endothelial cells and contribute to the vascular stabilization [16], [17], which are likely to act as a threshold role in the progression of diabetic microvascular complications in heart.

Increasing evidence also shows that that the prototype S1P receptor modulator FTY720, isolated from the ascomycete Isaria sinclairii, binds with high-affinity to S1P1 and S1P3 and acts differently depending on the receptor subtype and the targeted cell type/tissue [18]. A wide range of studies–from those on targeted tumor therapy to multiple sclerosis treatment–have been carried out on FTY720, suggesting a potent effect on vascular homeostasis [19]–[23]. Diverse signaling related to the function of FTY720 has been attributed, in part, to the activation of a family of protein kinase C (PKC) [24]. However, the role of a specific subtype has yet to be addressed.

Knowledge of whether and how FTY720 exerts an effect on cardiac microvascular modulation in diabetes can serve as a foundation for future investigations. Thus, current study is to provide detailed data on the cardiac microvascular effects of FTY720 and its possible mechanism related with S1P1/3 in diabetic heart disease. This work may disclose a novel therapeutic approach to improve cardiac function of patients with diabetes.

## Materials and Methods

### Animals for the *in vivo* experiment

In the experiment in vivo, to reduce the influence from blood glucose level and increase the feasibility of cardiac microvascular observation through vascular casting and lanthanum perfusion, the diabetic rat induced by STZ was adopted. The Committee of Ethics on Animal Experiments, The Forth Military Medical University, reviewed and approved this study protocol. All research was performed on 8-week-old Sprague-Dawley rats. We induced diabetes with a single intraperitoneal injection of streptozotocin (50 mg/kg in 0.9% saline) (Sigma). Only rats that developed sustained hyperglycemia with a serum glucose level >300 mg/dL were included in the study.

Diabetic rats were randomized to receive vehicle or FTY720 (Cayman Chemical, USA) administration (1 mg/kg) with intraperitoneal injection for 8 weeks. Age-matched rats were used as non-diabetic controls (n = 15 in each group). The animals were maintained in a room controlled at a temperature and relative humidity of 24±28° C and 40–70%, respectively. Food and water were provided *ad libitum*. And the serum glucose of all groups was tested every 2 weeks during the experiment.

### Echocardiography

Transthoracic echocardiography was performed with a 16.0- to 21.0-MHz transducer (VEVO 2100, VisualSonics). LV end-diastolic (LVEDD) and end-systolic (LVESD) dimensions were measured according to the modified American Society of Echocardiography– recommended guidelines. Fractional shortening (FS) and cardiac output (CO) were calculated according to pervious study[10]. All measurements represent the mean of at least 3 consecutive cardiac cycles.

### Pressure Measurements

At established time points (0 week and 8 weeks after diabetes induced), systolic blood pressure was measured by the method followed. After proper anesthesia (single intraperitoneal injection with 30 mg/kg Pentobarbital Sodium), a small incision was made on the left cervical part, and carotid artery was dissected. Subsequently, a 1.4F high-fidelity pressure transducer (Micro-tip catheter, Millar Instrument) was introduced into LV along the artery. When hemodynamic stable, LV systolic pressure, end-diastolic pressure, and the maximal rates of pressure rise (+dP/dt) and of pressure fall (−dP/dt) were recorded with Power Lab System (AD Instruments).

### Transmission electron microscopy (TEM) detection *in vivo*

We used a transmission electron microscope with lanthanum tracer to determine the microvascular ultrastructure as well as permeability according to previous studies [11], [12], [25]. A peristaltic pump maintained blood pressure at 100 mmHg while two groups of anaesthetized rats were perfused transaortically with 50 ml of a prefixative solution (100 mM Tris, pH 7.2, 150 mM NaCl, 5.6 mM KCl, 1 mM MgCl2, 2.5 mM CaCl2, 3.7 mM glucose, and 3.6 mM procaine), followed by 250 ml of fixative (2.5% glutaraldehyde and 2% paraformaldehyde in 0.1 M sodium cacodylate buffer, pH 7.2, containing 2% lanthanum nitrate). Samples were fixed without lanthanum nitrate continuously for 1 hour and rinsed in washing solution (0.15 M NaCl plus 0.2 M sucrose). They were cut into ultrathin sections (60 nm thick) and mounted on copper grids (200 mesh) for examination under a transmission electron microscope (JEM-2000EX, Japan).

### Scanning electron microscopy (SEM) detection *in vivo*

Mercox (Company, Location) is a methylmethacrylate-based resin widely used for vascular corrosion casting with subsequent scanning electron microscopy[11], [26]. Rat hearts were removed and injected with 10 ml Mercox resin (SPI) (100 mmHg) through the aorta according to the manufacturer's instructions. The sample was incubated at room temperature for 1 hour, and 2 hours in a 60°C water bath, then dipped in a solution of NaOH hydrolysis and collagenase digestion for 2 weeks. We removed the connective tissue components surrounding the vasculature, and used regular pretreatments–including dehydration, desiccation and gilding–according to standard instructions. Then SEM observation was performed(Hitachi S-3400N, Japan).

### Tissue preparation

Four weeks after induction of diabetes, the rats were sacrificed, and their left ventricles sliced into 5-µm transverse/longitudinal sections. These were fixed with 4% paraformaldehyde or methanol for 18–24 hours, or frozen in OCT compound.

### Histological examination and morphometry

To investigate either apoptosis or pathological changes in cardiac microvessels, we used a dual labeling process with a double immunofluorescence sequential protocol. We performed two experiments. The first used anti-CD31 mouse monoclonal antibody (1∶100; Millipore, USA) as the primary antibody. We simultaneously used anti-S1P1 rabbit polyclonal antibody and anti-S1P3 rabbit polyclonal antibody (1∶100; Sigma, USA) as the second primary antibodies.

In a second experiment, we used anti- PKCβII rabbit polyclonal antibody (1∶200; ABcam, USA) as the primary antibody. FITC, TRITC, or HRP-conjugated secondary antibodies were respectively selected based on the primary antibody. In controls, the primary antibody was replaced with normal rabbit serum (DAKO, Denmark). We used ordinary or fluorescence microscopy (Olympus DP90, Japan) for imaging, and Adobe Photoshop CS5 for processing.

### Apoptosis determination by TUNEL

Cardiac microvascular apoptosis was detected by TUNEL (Promega, USA) and CD31 (1∶100; Millipore, USA) dual labeling in paraffin-embedded sections under a 200X magnification fluorescence microscope. The pretreatment of tissue sections–including deparaffinizing, rehydration, retrieval with reconstituted Proteinase K, equilibration, and slide washing–were performed according to manufacturer's instructions. Tissue slides were incubated with Nucleotide Mix and rTdT buffer solution at 37°C for 60 minutes to allow sufficient reaction, then terminated by 2X SSC solution for 15 minutes. The control incubation buffer was prepared without the rTdT enzyme; all other steps were similar. Samples were stained with DAPI to determine total nuclei number. We used CD31 (1∶100; Millipore, USA) immunofluorescence to identify endothelial cells. The same methods described above were applied. Samples were observed and immediately analyzed under a fluorescence microscope (Olympus DP90, Japan).

### Laser capture microdissection (LCM)[27]

Sections (5 µm thick) of the left ventricle were mounted on Junbo slides (Leica, Germany) and dewaxed in toluol (2×3 min). Slides were washed with isopropyl alcohol (2×1 min), 90% ethanol (1 min), 70% ethanol (1 min), and water (1 min), then rapidly stained with haematoxylin Carazzi (1 g/l, 20 s each). Sections were washed with water (1 min); dehydrated with an ethanol gradient (70% ethanol [1 min], 90% ethanol [1 min], 100% ethanol [2×1 min]); washed with xylene (5 min); and air-dried (20 min). Cardiac microvascular cells were microdissected using the Leica AS LMD system (Leica, Germany). LCM parameters were 30 µm spot size, 100 mW power, 4.8 mA current, 7.3 ms duration, and 0.2 s repeat time.

Isolation and identification of cardiac microvascular endothelial cells (CMECs) for the *in vitro* experiment.

The pathophysiology of cardiac vascular lesions associated with diabetes occurs in arterial vessels with a diameter <250 µm, emphasizing the necessity for well-characterized microvascular endothelial cell preparations. And the same method described below was used as previous studies[11], [28], [29]. Left ventricles of rats were harvested and minced into 1 mm^3^ pieces after removal of the endocardial endothelium and the epicardial coronaries from male Sprague–Dawley rats weighing 100–120 g. The tissue pieces were dissociated by collagenase II (1 mg/mL; Invitrogen, USA) and subsequently cultured in Endothelial Growth Medium consisting of defined growth factors and supplemented with additional FBS up to 15% final concentration. Passage 3–5 cells were used for further studies. The cells were identified by staining with 15 µg Alexa Fluor 594 AcLDL overnight at 4°C and CD31(Chemicon, USA). FTY720 (Cayman Chemical, USA) was dissolved in dimethyl sulfoxide (DMSO) (Sigma, USA) to a final concentration of 10 nmol/L. Afterwards, CMECs were cultured in different mediums, including high glucose (HG) (25 mmol), high glucose+FTY720 (10 nmol/L), and CMECs with PKCβII overexpressed in high glucose+FTY720.

### Lentivirus-based overexpression of PKCβII

We carried out lentivirus-based transduction using the packaging cell line HEK293T by cotransfection of the plasmid pReceiver-Lv105 alone (EX-EGFP-Lv105, Genecopoeia, USA) or pReceiver-Lv105 containing full-length PKCβII cDNA (EX-T0190-Lv105, Genecopoeia, USA) and the packaging plasmids (HPK-LvTR-20, Genecopoeia, USA) according to the manufacturer's protocols. CMECs were then infected with the lentivirus particles. Efficacy was assessed after 48 h by RT-PCR and Western blot.

### Migration assay *in vitro* by Transwell chambers

CMECs were suspended at 1×10^6^/mL in medium containing DMEM with 0.1% bovine serum albumin; 100 µL cell suspension was added to the upper well of Transwell inserts coated with 1 mg/mL Matrigel (BD Biosciences, USA). We added 0.6 ml medium in lower wells. The plates were incubated for 24 h at 37°C in 5% CO_2_. After incubation, the inserts were carefully lifted and cells from the upper surface gently removed. Remaining cells at the bottom side of the filter were fixed and stained with staining solution for 20 min. They were then counted and analyzed.

### RNA extraction and reverse transcription-PCR

Total RNA was extracted from microdissected untransfected or transfected CMEC tissues using TRizol (Invitrogen, USA) according to the manufacturer's instruction, and then reversely transcribed to prepare cDNA using the PrimeScript RT Reagent kit (DRR037S, TakaRa, Japan). The mRNA levels of S1P1, S1P3, and PKCβII were subsequently detected by PCR. The simultaneous amplification of GAPDH served as the control. Primers used are listed in Table 1.

**Table 1:** The primers used in this study.

Gene name	Accession No.	Primer sequence
S1P1	NM_017301	Sense: 5′- TTCTGCGGGAAGGAAGTATG -3′
		Antisense: 5′- TGCTGCCGTTGTGTAGTTTC -3′
S1P3	XM_225216	Sense: 5′- GGGAGGGCAGTATGTTCGTA -3′
		Antisense: 5′- AAGTTCTCCAGGCAGTTCCA -3′
PKCβII	NM_001172305	Sense: 5′-GAACTGACTCCCACTGACAAACT-3′
		Antisense: 5′-CACCATGAATCCTGGAAGACT-3′
GAPDH	NM_017008.3	Sense: 5′-GGAAAGCTGTGGCGTGAT-3′
		Antisense: 5′-AAGGTGGAAGAATGGGAGTT-3′

### Western blot of S1P1, S1P3, and PKCβII

We used Western blot analysis according to the manufacturer's instructions to investigate S1P1, S1P3, and PKCβ II protein expression. Samples were first homogenized in lysis buffer. Protein extracts (100 µg per sample) were separated on a 10% SDS-PAGE (Bio-Rad, USA), then electrotransferred onto PVDF membranes (Amersham Pharmacia). The membranes were incubated with anti-S1P1 rabbit polyclonal antibody(1∶500; Sigma, USA), anti-S1P3 antibdy (1∶500; Sigma, USA), or anti-PKCβII antibody (1∶500; ABcam, USA) overnight at 4°C with constant shaking. We confirmed equal protein loading by reprobing the membranes with a mouse monoclonal antibody to β-actin (1∶1000; Santa Cruz Technology, USA). The antibody-bound proteins were detected by chemiluminescence (ECF) (Amersham-Pharmacia Biotech, USA).

### Statistical analysis

All data were presented as means±SD. We performed ANOVA to compare the means of all groups of data. If the ANOVA indicated an overall significant difference (P<0.05), the pairwise multiple comparison test (Tukey or Dunn for morphology studies) was used to determine the significance of a difference in the means between any two groups; P<0.05 was considered significant.

## Results

### *In vivo* experiment

#### Recovery of cardiac function without blood glucose level alteration after FTY720 administration

**Figure 1:**
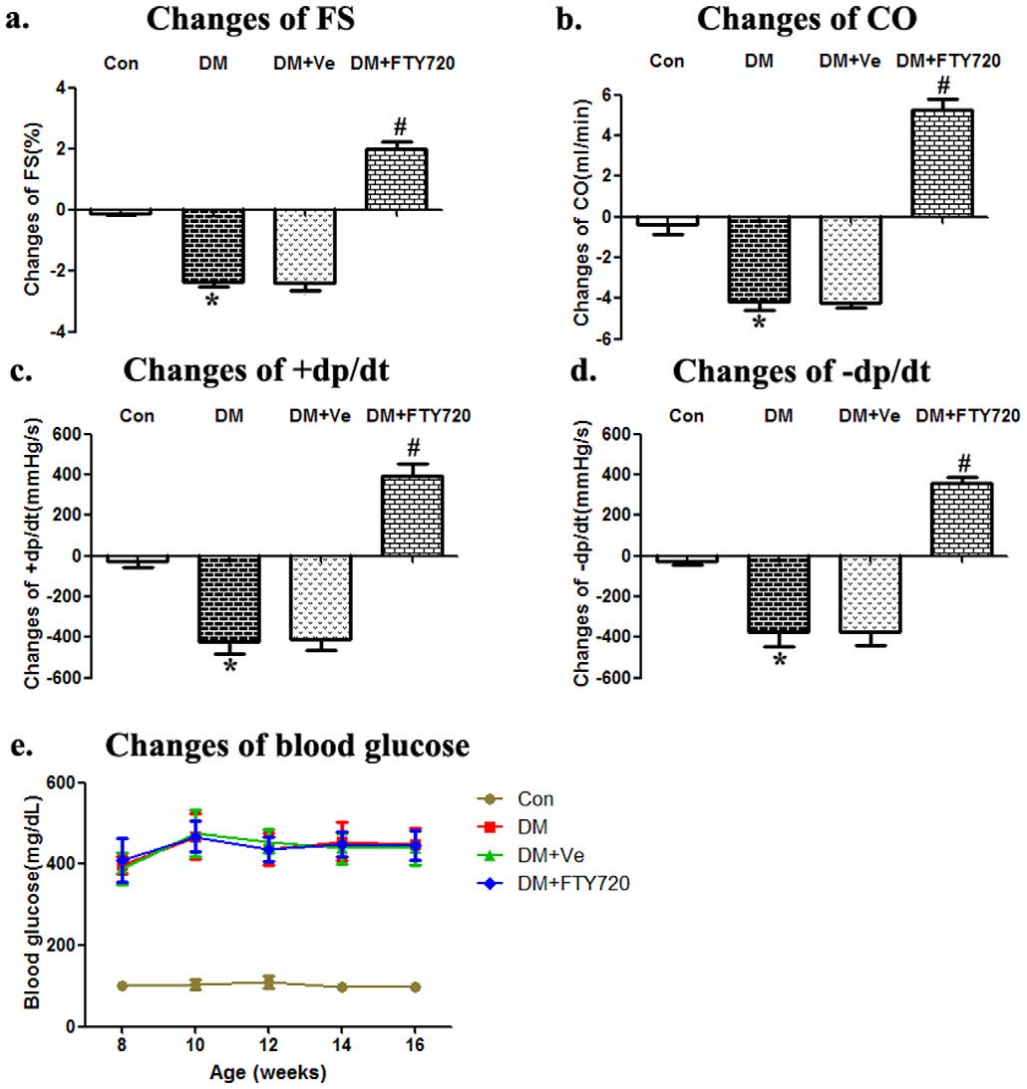
Cardiac function and blood glucose level analysis. Compared with Con group, the data, including FS, CO and ±dp/dt, revealed significantly decreased cardiac function of DM. And FTY720 could improve these changes. The group receiving vehicle alone did not exhibit any improvement (a–d). Fig. 1e showed all groups remained stable blood glucose level from the 8th week(Con<130 mg/dl; all diabetes >300 mg/dl). * P<0.05 Compared with the Control Group. # P<0.05 Compared with the DM Group.

Through echocardiography and micro-transducer LV pressure measurement, we evaluated the impact of FTY720 on global cardiac function. At baseline, compared with Con group, other groups (DM, DM+Ve, DM+FTY720) demonstrated significantly lower FS, CO and ±dp/dt (P<0.05; Table 2). After different treatment, cardiac function of DM group still continued decline during the 4 weeks follow-up period (DM vs. Con: FS, −2.17±0.16% *vs.* −0.12±0.07%, P<0.05; CO, −4.17±0.38 vs. −0.4±0.11, P<0.05; +dp/dt, −420±61.1 vs. −28.3±17.8, P<0.05; −dp/dt, −373.3±72.2 vs. −23.3±18.3, P<0.05; Fig. 1a–d). And FTY720 administration could improve global cardiac function (FTY720 vs. DM: FS, 1.97±0.24% *vs.* −2.17±0.16%, P<0.05; CO, 5.27±0.55 vs. −4.17±0.38 , P<0.05; +dp/dt 393.3±58.1 vs. −420±61.1, P<0.05; −dp/dt, 360.0±30.6 vs. −373.3±72.2, P<0.05; Fig.1a–d).

**Table 2:** Baseline Characteristics of Cardiac Function at 0 week after diabetes induced.

	Con	DM	DM+Ve	DM+FTY720
FS, %	37±2	25±3*	25±2*	26±2*
CO, ml/min	100±5	55±3*	53±6*	55±5*
+dp/dt, mmHg/s	4830±216	3850±320*	3900±318*	3886±299*
−dp/dt, mmHg/s	3816±168	2836±139*	2782±178*	2819±159*

* P<0.05 vs. Con.

Furthermore, to rigorously determine the effects of FTY720 on cardiac microvessels, blood glucose level was tested once every two weeks. And all rats remained stable blood glucose level from the 8th week (Con<130 mg/dl; all diabetes >300 mg/dl; Fig. 1e), which suggested FTY720 having no significant influences on the blood glucose level of STZ-induced diabetes.

#### Morphous and barrier function of diabetes were improved by FTY720 administration

Morphological information on the wall cells (including smooth muscle and endothelial cells) may facilitate our understanding of pathophysiological cardiac microvasculature. Therefore, we described the segmental constitution of microvessels and discussed the possible involvement of both permeability and angiogenesis based primarily on TEM and SEM observations.

**Figure 2:**
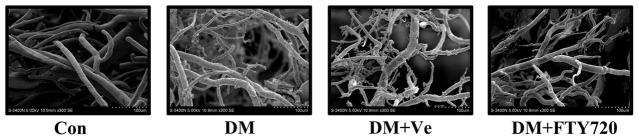
Angiogenesis observation 300x. The surfaces of cardiac microvascular walls were highly irregular in DM, with numerous evaginations and invaginations. FTY720 treatment attenuated these pathologic changes, preserving CMECs integrity and microvascular integration. The group that received vehicle alone did not exhibit any obvious changes compared with the DM group.

Direct observation, made possible by the scanning electron micrograph, has enabled us to extend our functional understanding of microvessels. The cardiac capillaries of rats with diabetes mellitus (DM) showed significantly higher density, and their arrangement was far more disordered and twiggy than those of the control group, which had brawny capillaries arranged in symmetrical, regular arrays (Fig. 2). The surfaces of cardiac microvascular walls were highly irregular in DM, with numerous evaginations and invaginations. Their configuration and size varied considerably, which might account for the loss of endothelial cells. FTY720 treatment attenuated these pathologic changes, preserving CMEC integrity and microvascular integration. The group that received vehicle alone did not exhibit any improvement compared with the DM group.

**Figure 3:**
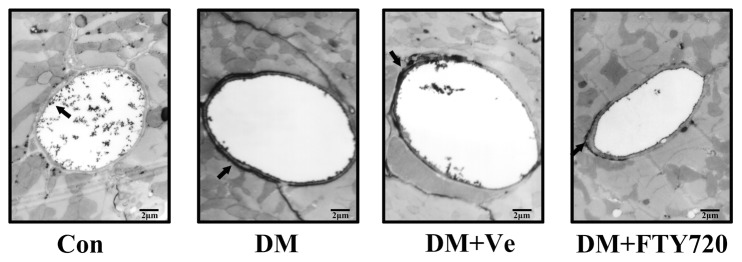
Cardiac microvascular permeability observation 1500x. The dark material directed by arrow, lanthanum, is unable to pass through normal microvascular wall. We observed distinguished lanthanum laver throughout the length of the inter-endothelial clefts and on the albuminal surface in DM cardiac microvessels, showing not only increased permeability, but also a significant destruction of microvascular wall. Comparable loss of barrier function was observed in the vehicle group as well as the DM group. In the latter, FTY720 improveed impairment of cardiac microvascular permeability.

Lanthanum is unable to pass through normal microvascular wall [30]. In all heart samples, lanthanum markers showed an even, electron-dense layer on the microvascular luminal surface (Fig. 3). However, DM cardiac microvessels had distinguished lanthanum laver throughout the length of the inter-endothelial clefts and on the albuminal surface, suggesting not only increased permeability, but also significant destruction of the microvascular wall. A comparable loss of barrier function was observed in both the vehicle and DM groups. However, administration of FTY720 improved dysfunction of cardiac microvascular permeability in diabetes.

#### FTY720 reduced the apoptosis of vascular endothelial cells in diabetes

**Figure 4:**
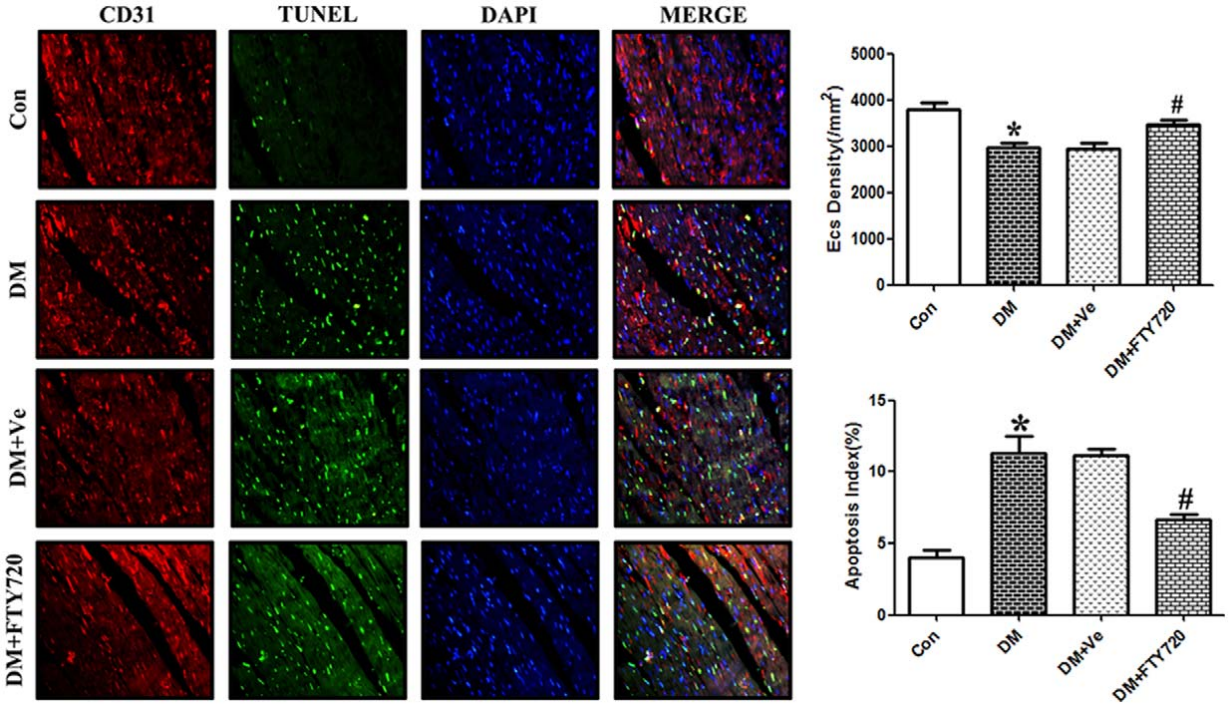
Count and apoptosis analysis of vascular endothelial cells 200×(CD31: Red; TUNEL: Green; DAPI: Blue). CD31 positive endothelial cells in diabetic rats were significantly decreased (P<0.05). The apoptosis index of cardiac endothelial cells in DM rats was much higher than in controls (P<0.05). FTY720 significantly slowed the apoptosis rate (P<0.05), while the vehicle group did not exhibit any difference with DM. * P<0.05 Compared with the Control Group. # P<0.05 Compared with the DM Group.

Figure 4 shows capillary density change and cardiac microvascular apoptosis in diabetic rats after double staining with TUNEL and CD31. CD31 positive endothelial cells were significantly reduced in the DM rats compared with controls (2800±113 vs. 3825±155, P<0.05). The apoptosis index (TUNEL-positive rate) of cardiac endothelial cells in DM rats was significantly higher than in the control group (11.7±0.9% vs. 3.8±0.8%, P<0.05). FTY720 significantly decreased the apoptosis rate (6.7±0.6% vs. 3.8±0.8%, P<0.05), with no difference between the DM and vehicle groups.

#### FTY720 increased the expression of S1P1 and PKCβII, decreased the expression of S1P3 in diabetic cardiac microvessels

**Figure 5:**
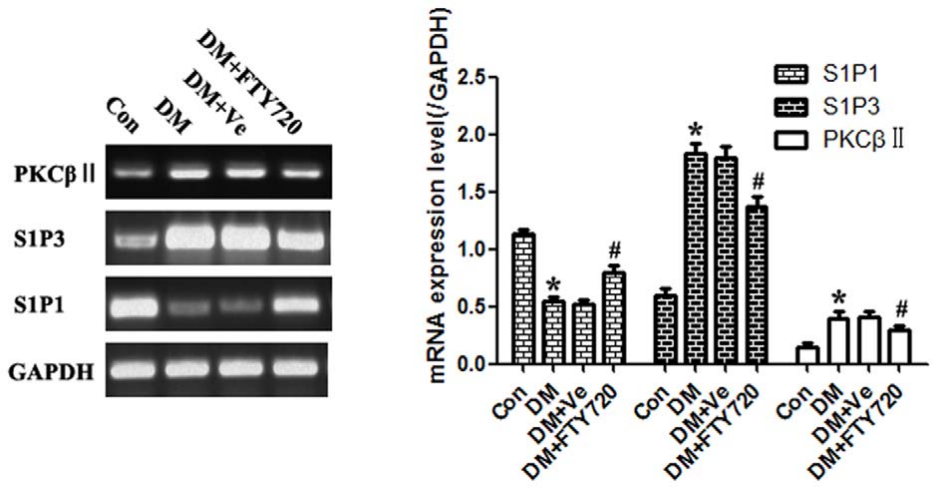
S1P1, S1P3, and PKCβII mRNA expression analysis of cardiac microvessels. Up-regulation of S1P3 and PKCβII or down-regulation of S1P1 in DM differed significantly from controls (P<0.05). Regulation of S1P1, S1P3, and PKCβII was reversed in the DM+FTY720 group with administration of FTY270 (P<0.05). There was no significant difference between the vehicle and DM groups. * P<0.05 Compared with Control Group. # P<0.05 Compared with DM Group.

Fig. 5 shows the relationship between cardiac microvascular pathology and S1P1, S1P3, or PKCβII at mRNA level after LCM. Compared with controls, our finding showed a significant difference in up-regulation of S1P3 and PKCβII or down-regulation of S1P1 in DM (P<0.05). FTY720 administration reversed changes of S1P1 and S1P3. Interestingly, FTY720 also made obvious influence on decreasing the expression of PKCβII in (DM+FTY720) group (P<0.05), suggesting PKCβII might be a key factor on the signaling pathway, through which FTY720 exert its effect. And no significant difference was observed between the vehicle and DM groups.

However, as an agonist on S1P1/3, FTY720 down-regulating the expression of S1P3 made us confused. And in consideration of 5 µm thick slides collected by microdissection can not obtain integrated cell structure, especially the whole nuclear, there might leave out important information in vivo experiment. Thus, we decided to continue the detection *in vitro* experiment to explain the question about FTY720′s effect on S1P3.

### *In vitro* experiment

#### FTY720 stimulated the migration of CMECs in high glucose medium

**Figure 6:**
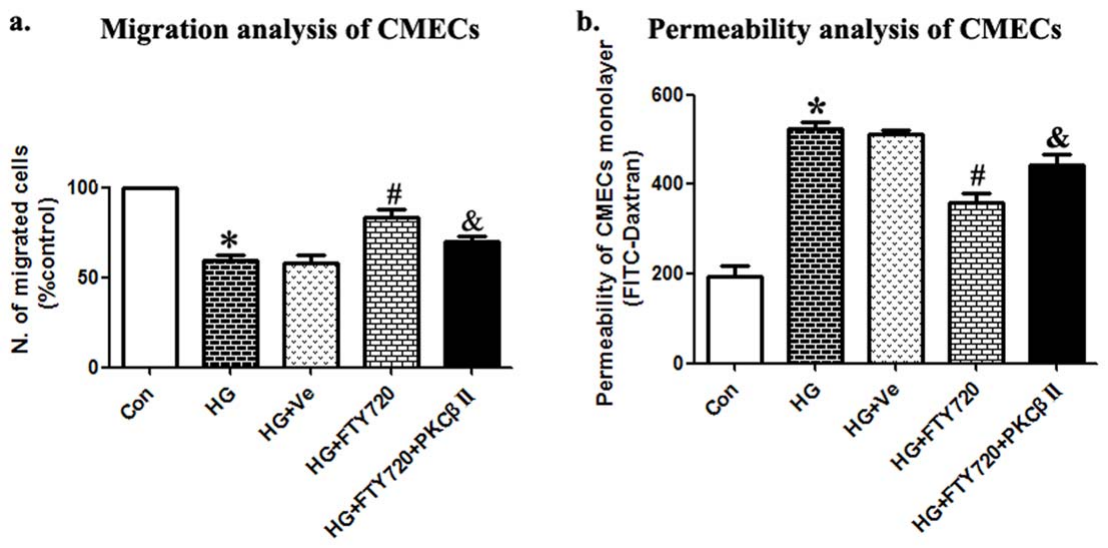
Migration and permeability analysis of CMECs. a. Administration of FTY720 in medium almost reversed migration induced by high glucose to a normal state (P<0.05). However, PKCβII up-regulation suppressed the effect of FTY720 (P<0.05), suggesting the involvement of PKCβII in FTY720-induced CMEC migration.b. Relative fluorescence level unit (RFU) was higher when performed in high glucose compared with controls (P<0.05). This suggested increased leakage of monolayer CMECs. FTY720 could attenuate the permeability change induced by high glucose (P<0.05), and in the PKCβII overexpression group, the effect of FTY720 was significantly decreased (P<0.05). * P<0.05 Compared with Control Group. # P<0.05 Compared with HG Group. & P<0.05 Compared with HG+FTY720 Group.

CMECs were successfully cultured and identified in our previous study [11], and overexpression of PKCβII based on Lentivirus was assessed as successful. In the present study, we used Transwell assays to test migration, a key step in angiogenesis. The data showed that administration of FTY720 in medium reversed migration induced by high glucose to almost a normal state (78.0±4.30% vs. 61.5±2.80%, P<0.05). However, up-regulation of PKCβII suppressed the effect of FTY720 (68.0±3.00% vs. 78.0±4.30%, P<0.05), suggesting the involvement of PKCβII in FTY720-induced CMEC migration (Fig. 6a).

#### FTY720 help barrier function recovering of monolayer CMECs

The In Vitro Vascular Permeability Assay kit is considered an accurate method of assessing monolayer cell permeability. FITC-Dextran clearance and fluorescence analysis showed a higher relative fluorescence unit (RFU) in high glucose medium than in the control group (529.0±7.00 vs. 190.0±20.00, P<0.05), suggesting increased leakage of monolayer CMECs. FTY720 attenuated the permeability change induced by high glucose (373.0±20.00 vs. 529.0±7.00, P<0.05). However, the effect of FTY720 was significantly decreased in the PKCβII overexpression group (485.0±20.00 vs. 373.0±20.00, P<0.05) (Fig. 6b).

#### FTY720 reduced the apoptosis index of CMECs

**Figure 7:**
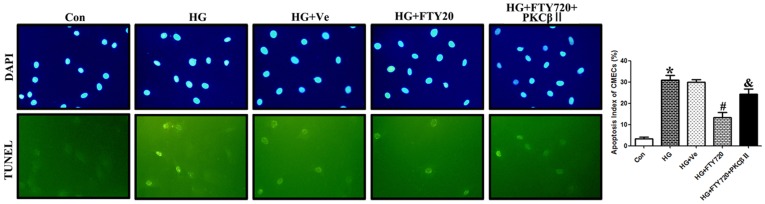
Apoptosis analysis of CMECs with double immunofluorescence 200x (TUNEL: Green; DAPI: Blue). The HG and HG+Ve groups had a higher apoptosis index compared with controls (P<0.05). FTY720 could normalize the index (P<0.05). When PKCβII was up-regulated, the anti-apoptosis effect of FTY720 was significantly significantly decreased(P<0.05). * P<0.05 Compared with Control Group. # P<0.05 Compared with HG Group. & P<0.05 Compared with HG+FTY720 Group.

Double staining (TUNEL, green; DAPI, blue) revealed a higher apotosis index in the HG group (30.0±5.000% vs. 1.73±0.305%, P<0.05). FTY720 normalized the index (12.0±6.00% vs. 30.0±5.00%, P<0.05). Overexpression of PKCβII significantly decreased the anti-apoptosis effect of FTY720 (25.0±6.00% vs. 12.0±6.00%, P<0.05) (Fig. 7).

#### FTY720 decreased the expression of S1P1 & PKCβII, and boosted the translocation of S1P3

Compared to controls, a significant decrease of S1P1 and increase of PKCβII was demonstrated in endothelial cells exposed to high glucose medium (P<0.05), which were coincident with the results of *in vivo*. Besides, there appeared an interesting phenomenon in HG medium that S1P3 was down-regulated on cellular nuclear and up-regulated on membrane (P<0.05), which suggested the translocation of S1P3 from nucler to membrane.

On the other side, FTY720 not only attenuated the expressional changes of S1P1, but also reversed the translocation of S1P3 induced by the high glucose, indicating the different role of FTY720 on S1P1 or S1P3 (n = 15, P<0.05). And the expression level of PKCβII was decreased by FTY720 as well as in vivo.

**Figure 8:**
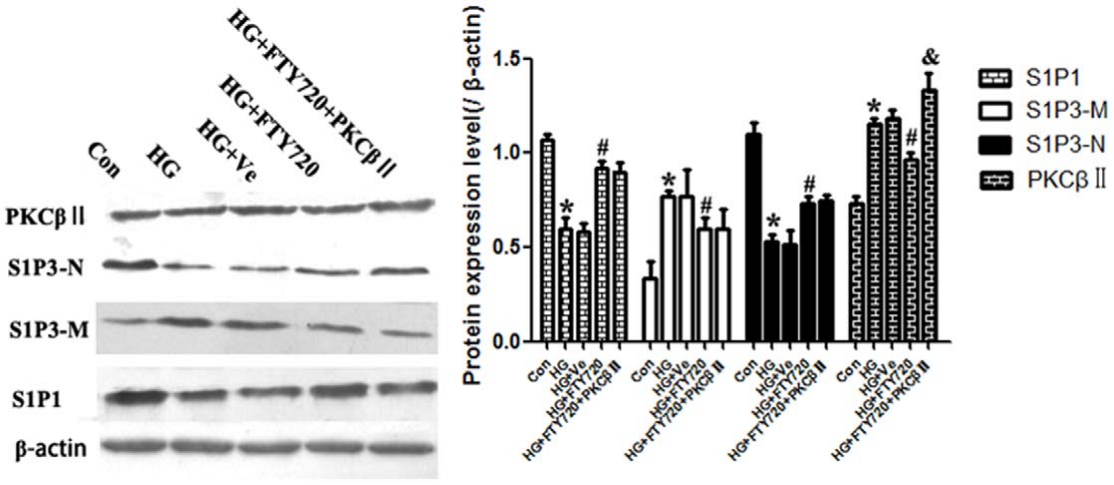
S1P1, S1P3, and PKCβII protein expression analysis of CMECs. Compared with control, we detected a significant decrease of S1P1 and increase of PKCβII in HG group. Meanwhile, a coincidence of increased membrane and decreased nuclear expression of S1P3 was found, suggesting a S1P3 translocation induced by glucose (P<0.05). FTY720 attenuated the changes of S1P1 and PKCβII (P<0.05), and reverse the translocation of S1P3. However, when PKCβII overexpressed, there were no significant changes in S1P1 and S1P3 compared with the HG+FTY720 group, indicating no feedback from PKCβII to S1P1/3 exist. * P<0.05 Compared with Control Group. # P<0.05 Compared with HG Group. & P<0.05 Compared with HG+FTY720 Group.

Subsequently, we observed, compared with (High Glucose+FTY720) group, no significant changes of S1P1 and S1P3 in PKCβII overexpression group. These suggested that although FTY720 regulating S1P1/3 might affect PKCβII, there existed no passive feedback from PKCβII to S1P1/3 (Fig. 8).

## Discussion

Through *in vivo* and *in vitro* experiments, present study addresses firstly that 1) As an important factor of diabetic heart disease, cardiac microangiopathy is characterized by increased vascular endothelial cellular apoptosis, elevated permeability and pathological angiogenesis; 2) Deregulation of S1P1 and S1P3 is an important signal responsible for cardiac microvascular complications in diabetes; 3) FTY720 could improve cardiac function-related microvascular conditions through up-regulating the expression of S1P1 and boosting the translocation of S1P3; 4) FTY720′s contribution to cardiac microvascular homeostasis is at least partly dependent on PKCβII-related signaling pathway.

As other ischemic events, our findings demonstrate that coronary collateral blood vessel formation in diabetes is an attempt to preserve cardiac function and myocardial viability by reducing myocardial ischemia and functional deficit. However, the increased cardiac microvessels are considered as a cardinal feature of pathological angiogenesis in DM [31]. And the reasons lie in that the newly-formed microvessels induced by hyperglycemia and other persistent stimuli are too leaky, too complex and nonfunctional, resulting in the decreased and slowed cardiac blood supply. To elucidate the biological basis for the pathological angiogenesis in diabetic heart, we investigated the retarded migrating ability and ascendant permeability of cardiac microvascular endothelial cells in high glucose medium. Under physiological condition, the properties of permeability and migration complement each other to support normal angiogenic process [31]–[33]. In diabetes, the increase of permeability stimulates endothelial cells sprouting to promote angiogenesis, however, which cannot be kept pace with by weakened ability of migration. This could explain the fact that cardiac microvessels in diabetes are not well organized and are not completely covered with an intact layer showed in scanning electron micrograph. Finally, this contradistinctive change of migration and permeability is considered to accelerate and aggravate the whole pathological process of cardiac microvessels. Furthermore, it is found that the morbidness of microvessels is substantially caused by dysfunction of the endothelial cells, which are thought to maintain the permeability and integrity of the vessel wall [34]. The possible mechanisms involved include cellular apoptosis, mechanical injury, decreased survival of cytoskeletal proteins, alteration of endothelial cellular adhesion molecules, and defective binding to anchoring matrix proteins [11], [12]. In our study, we found increased apoptosis of endothelial cells and fewer CD31 positive endothelial cells due to either necrosis or endothelial cell detachment from the neoformative pathological microvessels. The interaction between endothelial cells and membrane proteins of the microvessels is weakened by persistent apoptosis, necrosis, and cellular detachment , leading to a state of de-endothelialization, with the “sloughed off” vessel walls indicating severe endothelial damage and microvascular dysfunction. Hence, despite of the excessive angiogenesis, the cardiac microvascular supply for the myocardium still remains insufficient. This contradiction leads to a cascade starting from microvascular damage to deteriorative cardiac function.

S1P, a bioactive lipid,plays a crucial role in cardiovascular system, such as modulating the proliferation, differentiation, migration, and survival of endothelial cells, and affecting smooth muscle or bone marrow cells via activation of the G protein-coupled S1P receptors [35]. Surely, most vascular endothelial cells are now known to express S1P1 and S1P3 (but not other subtypes), suggesting that these two receptors are the main mediators of S1P-related action on cardiac microvascular endothelial cells. In particular, previous studies indicate that activation of S1P1 emphasize on promoting angiogenesis, whereas stimulation of S1P3 lead to impariment of barrier function [36]. Our study found accompany with increased cardiac microvascular permeability and pathologic angiogenesis in diabetes, S1P1 was down-regulated and S1P3 was translocated from nuclear to membrane, which were in agreement with the role of S1P1/3 mentioned above. These outcomes point to the existence of a passive feedback reaction in diabetic cardiovascular disease, suggesting that the deregulation of S1P1 and S1P3 may be an important signal responsible for cardiac microvascular complications in diabetes. Monitoring at the alterations of S1P1 and S1P3 could not only represent the cardiac microangiopathy like other molecules, but also respectively reflect the condition of microvascular permeability and pathological angiogenesis in diabetic heart.

FTY720 is derived from myriocin, a fungal metabolite used in traditional Chinese medicine. So far, it is the only specific agonist that acts on S1P1 and S1P3 [24]. As a pleiotropic mediator, it has not been addressed whether and how FTY720 could exert its therapeutic potential on cardiac microvascular dysfunction in diabetes. Our *in vivo* and *in vitro* experiments showed that in diabetic heart, FTY720 exert its agonism by up-regulating S1P1, and exert its functional antagonism by stimulating the translocation of S1P3 from cellular membrane to nuclear. For one thing, the functional discrepancy of FTY720 on S1P1 and S1P3 may depend on the particular characteristics of CMECs [28] ; For another, it may relate to a differential association of the receptor with specific molecules in lipid rafts or the stations where G protein-coupled receptors (GPCRs) accomplish specific signaling and internalization/recycling tasks [37]. Similarly, FTY720′s down-modulation on S1P1 but not S1P3 is showed on T cells during adaptive immune responses[38]. FTY720 could internalize and degrade membrane S1P1 in lymphocytes to prevent egress from lymph nodes and recirculation to peripheral inflammatory tissues[39], [40]. This unique effect is helpful to prevent acute immunological rejection and reduce severe infection caused by low immunity in organ transplantation. To be speculated, it may provide an opportunity against diabetic infection [18], [41], [42], which still need to be further research confirmed. At last, FTY720 elicits a variety of functional effects on cardiac microvessels, including reducing apoptosis of CMECs, enhancing cardiac microvascular permeability and improving angiogenesis. In addition, previous studies have showed that FTY720 could exert its immunesuppression on pancreas to reduce blood glucose level of diabetic mice [41], [43]. In present study, we used another diabetic model induced by STZ, and didn't found FTY720 with any influence on blood glucose level. In our consideration, this discrepancy may be derived from the different process of model construction. However, the stable blood glucose level of STZ-induced model is more beneficial to focus on the direct action of FTY720 in diabetic heart. And without consideration of FTY720′s potential on pancreas *in vivo*, our experiment *in vitro* provided more certainty on the cardiac microvascular protection of FTY720.

Thus, FTY720′s coupling of two S1P receptor subtypes (S1P1 and S1P3) to different functions, in the regulation of vascular endothelial barrier integrity and angiogenic homeostasis in cardiac microvasculature, might provide a molecular substrate to the complex modulation of angiogenesis. On one side, the different effects of FTY720 on S1P1 and S1P3, which is not ordinary up/down-regulation, demonstrated the specific advantage of pharmacological approach. On the other side, this effect of FTY720 on cardiac microvascular endothelial cell in diabetes is comparable to that of VEGF. Although VEGF and its receptors have a positive regulating effect on permeability or angiogenesis, they are still not competent enough to resolve the problem of pathologic angiogenesis, such as aberrant permeability and non-functional microvascular growth in diabetes [44], [45]. Thus, delivery of VEGF in the presence of diabetes needs to be carefully approached. Our study demonstrates that FTY720 may act as a agonist for S1P1 but functional antagonist for S1P3, which could help to keep the balance between microvascular barrier function and angiogenesis. Of course, further mechanistic insights will be required to confirm the details and characterize the pathways between FTY720 and VEGF, which is beneficial to alleviate cardiac microangiopathy in diabetes.

For more, our prior study has proved PKCβII making great contributions to the development of cardiac microangiopathy induced by diabetes [11]. And present study found that through regulating S1P1/3, FTY720 could decrease the expression of PKCβII at either mRNA or protein levels. Experiment *in vitro* showed that the overexpression of PKCβII could weaken the effects of FTY720 on CMECs, including migration, permeability and apoptosis, but without any significant influence on S1P1 and S1P3. These data suggest a unidirectional relationship from FTY20 to PKCβII, that FTY720 exerting its effects partly depends on PKCβII, but without passive feedback from PKCβII to S1P1/3. This finding is consistent with our prior study on PKCβII [11], and confirms a greater influence of FTY720 on cardiac microvascular endothelial cells in diabetes.

Our data demonstrate the important and novel finding that FTY720 might be competent to serve as a potential therapeutic approach for diabetic heart disease. Nevertheless, serious cardiac side effects, such as fatal bradyarrhythmia, induced by FTY720 suggest this treatment remains a major challenge [46], [47]. On the other side, several studies have clearly defined a protective role of FTY720 on heart through relieving either bradyarrhythmias or tachyarrhythmias caused by cardiac ischemic/reperfusion injury [47]. In addition, it also implies the potency in medication safety in a long-term phase III clinical trial for FTY720′s treatment on autoimmune disease and organ transplantation [24], [48], [49]. Accordingly, there still need further studies to determine the proper usage of FTY720.

Taken together, our findings not only firstly defined an important role of S1P1/3 in cardiac microvascular pathogenesis, but also revealed the unique treatment of FTY720 via S1P1/3-PKCβII -dependent pathway. This would provide a novel conceptive foundation for protecting cardiac microvessels, which may retard or prevent the deterioration of cardiac function of patients with diabetes.
